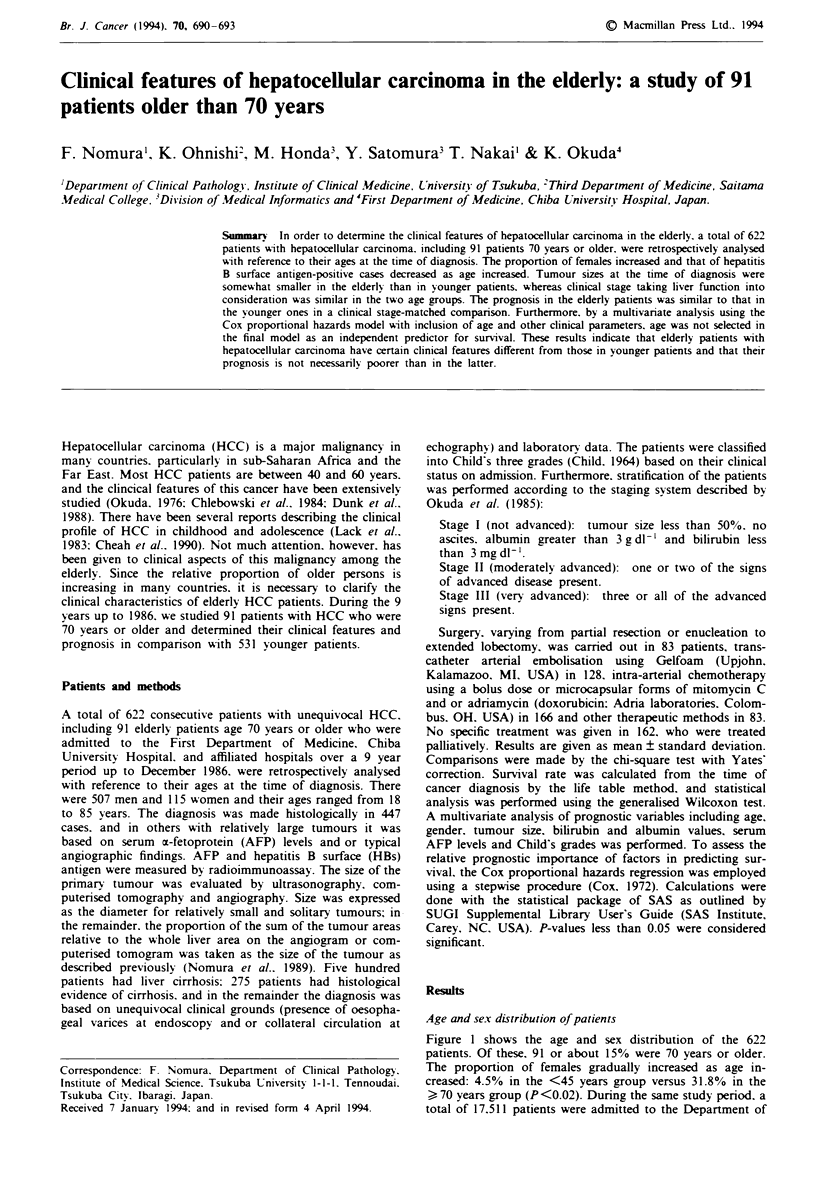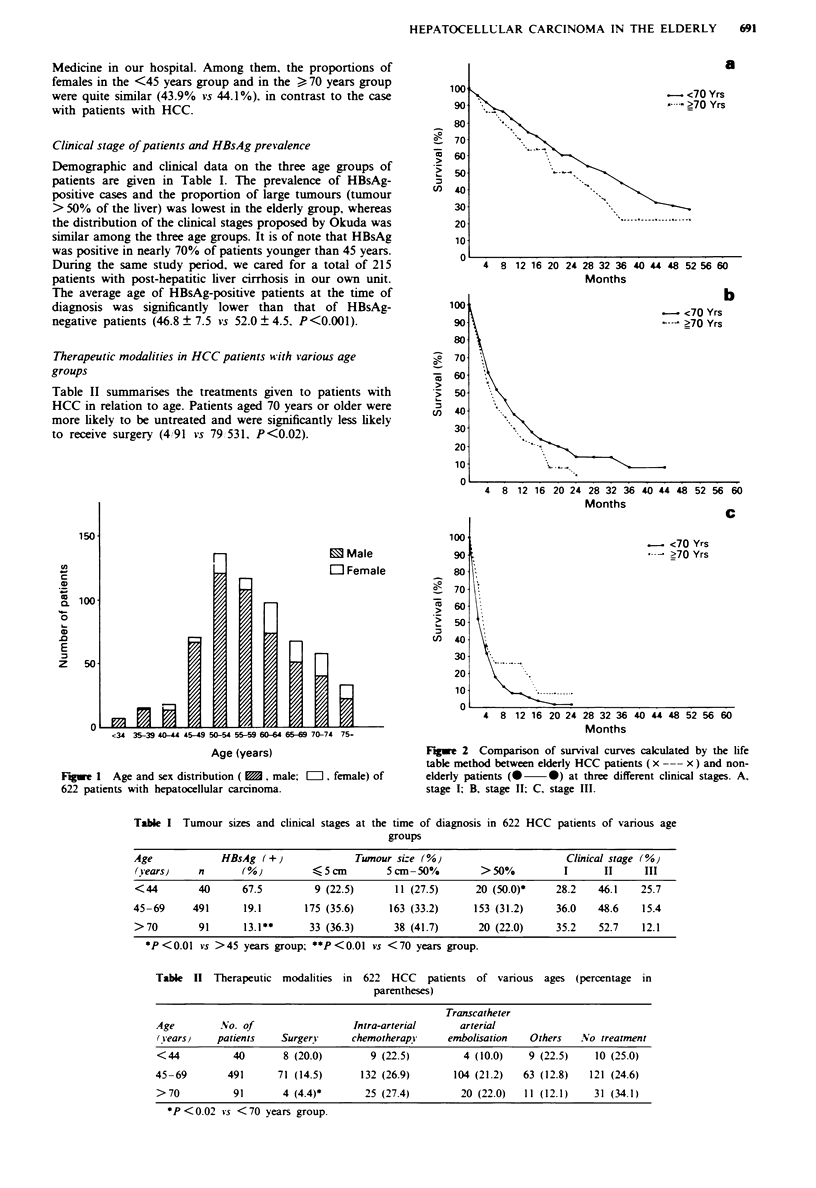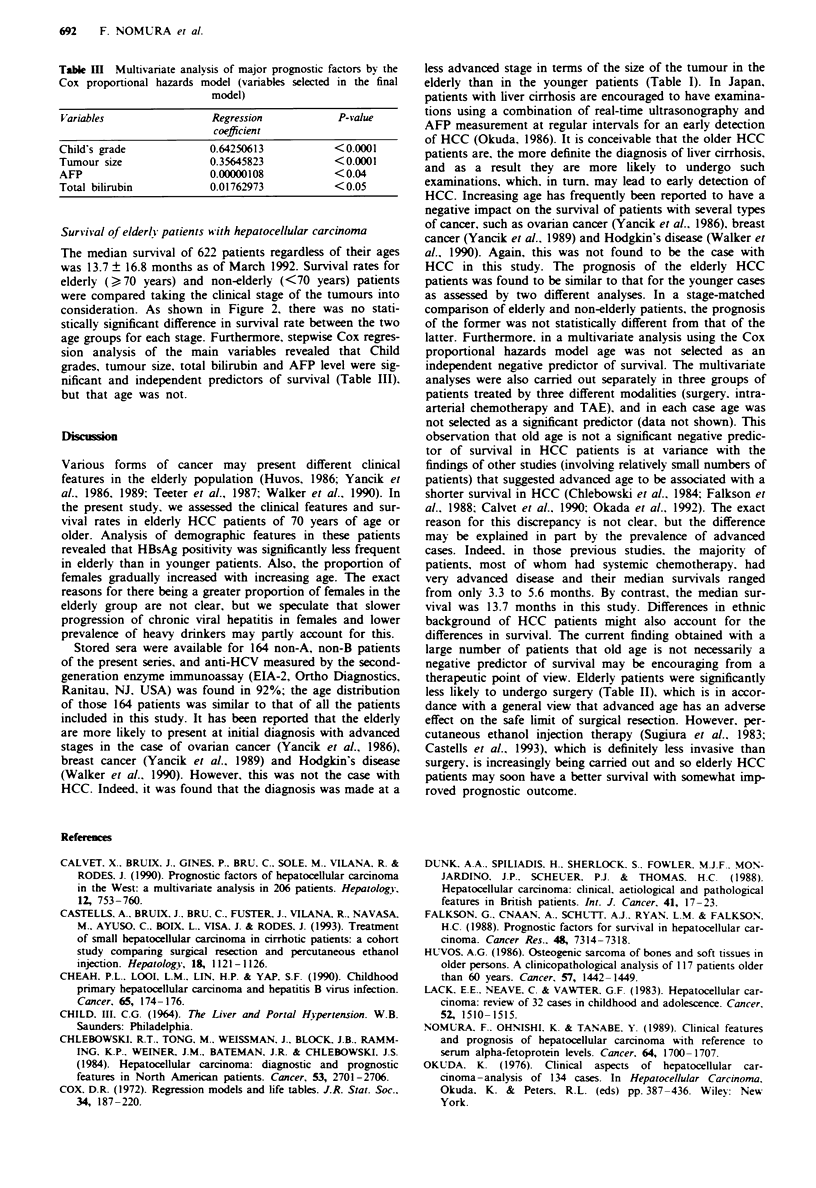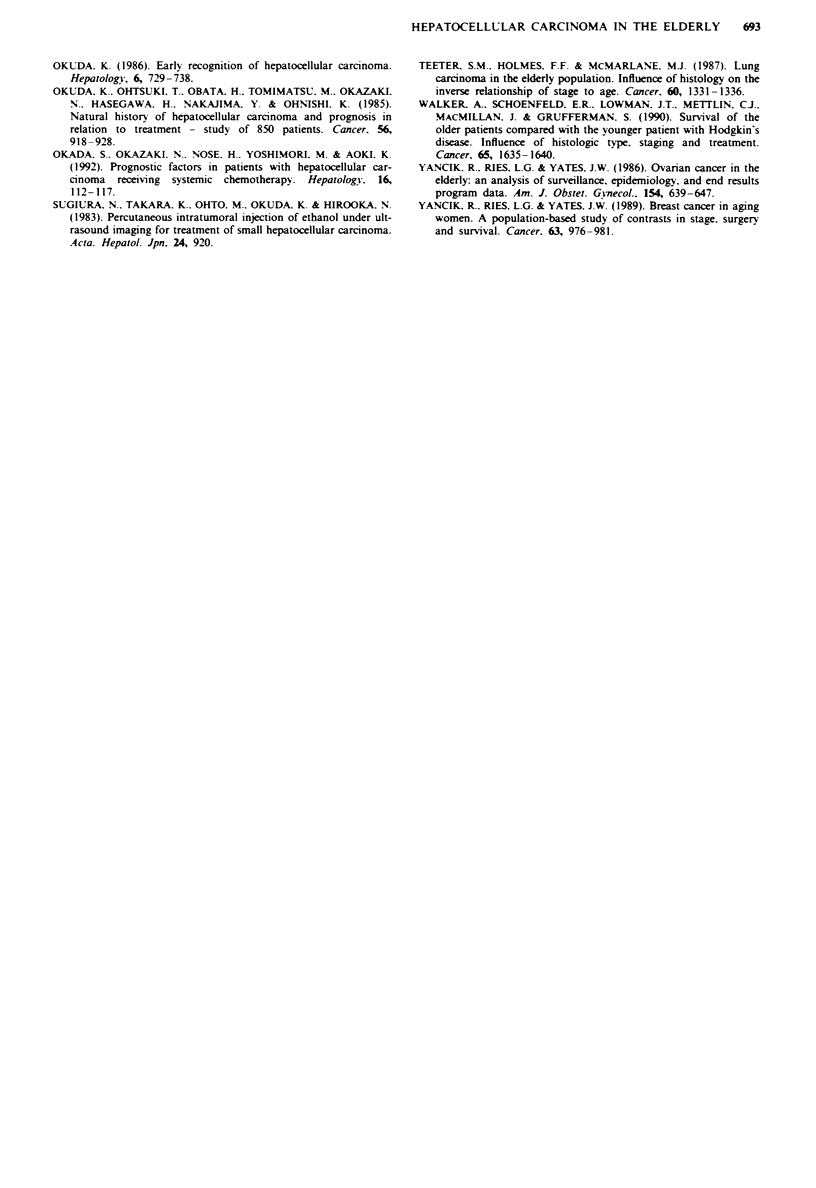# Clinical features of hepatocellular carcinoma in the elderly: a study of 91 patients older than 70 years.

**DOI:** 10.1038/bjc.1994.374

**Published:** 1994-10

**Authors:** F. Nomura, K. Ohnishi, M. Honda, Y. Satomura, T. Nakai, K. Okuda

**Affiliations:** Department of Clinical Pathology, University of Tsukuba, Japan.

## Abstract

In order to determine the clinical features of hepatocellular carcinoma in the elderly, a total of 622 patients with hepatocellular carcinoma, including 91 patients 70 years or older, were retrospectively analysed with reference to their ages at the time of diagnosis. The proportion of females increased and that of hepatitis B surface antigen-positive cases decreased as age increased. Tumour sizes at the time of diagnosis were somewhat smaller in the elderly than in younger patients, whereas clinical stage taking liver function into consideration was similar in the two age groups. The prognosis in the elderly patients was similar to that in the younger ones in a clinical stage-matched comparison. Furthermore, by a multivariate analysis using the Cox proportional hazards model with inclusion of age and other clinical parameters, age was not selected in the final model as an independent predictor for survival. These results indicate that elderly patients with hepatocellular carcinoma have certain clinical features different from those in younger patients and that their prognosis is not necessarily poorer than in the latter.


Br. J. Cancer (1994), 70, 690-693 © Macmillan Press Ltd., 1994

### Clinical features of hepatocellular carcinoma in the elderly: a study of 91 patients older than 70 years

F. Nomura[1], K. Ohnishi[2], M. Honda[3], Y. Satomura[3] T. Nakai[1] & K. Okuda[4]

[1]*Department of Clinical Pathology, Institute of Clinical Medicine, University of Tsukuba,* [2]*Third Department of Medicine, Saitama Medical College,* [3]*Division of Medical Informatics and* [4]*First Department of Medicine, Chiba University Hospital, Japan.*

**Summary** In order to determine the clinical features of hepatocellular carcinoma in the elderly, a total of 622 patients with hepatocellular carcinoma, including 91 patients 70 years or older, were retrospectively analysed with reference to their ages at the time of diagnosis. The proportion of females increased and that of hepatitis B surface antigen-positive cases decreased as age increased. Tumour sizes at the time of diagnosis were somewhat smaller in the elderly than in younger patients, whereas clinical stage taking liver function into consideration was similar in the two age groups. The prognosis in the elderly patients was similar to that in the younger ones in a clinical stage-matched comparison. Furthermore, by a multivariate analysis using the Cox proportional hazards model with inclusion of age and other clinical parameters, age was not selected in the final model as an independent predictor for survival. These results indicate that elderly patients with hepatocellular carcinoma have certain clinical features different from those in younger patients and that their prognosis is not necessarily poorer than in the latter.

Hepatocellular carcinoma (HCC) is a major malignancy in many countries, particularly in sub-Saharan Africa and the Far East. Most HCC patients are between 40 and 60 years, and the clincical features of this cancer have been extensively studied (Okuda, 1976; Chlebowski *et al.*, 1984; Dunk *et al.*, 1988). There have been several reports describing the clinical profile of HCC in childhood and adolescence (Lack *et al.*, 1983; Cheah *et al.*, 1990). Not much attention, however, has been given to clinical aspects of this malignancy among the elderly. Since the relative proportion of older persons is increasing in many countries, it is necessary to clarify the clinical characteristics of elderly HCC patients. During the 9 years up to 1986, we studied 91 patients with HCC who were 70 years or older and determined their clinical features and prognosis in comparison with 531 younger patients.

### Patients and methods

A total of 622 consecutive patients with unequivocal HCC, including 91 elderly patients age 70 years or older who were admitted to the First Department of Medicine, Chiba University Hospital, and affiliated hospitals over a 9 year period up to December 1986, were retrospectively analysed with reference to their ages at the time of diagnosis. There were 507 men and 115 women and their ages ranged from 18 to 85 years. The diagnosis was made histologically in 447 cases, and in others with relatively large tumours it was based on serum α-fetoprotein (AFP) levels and or typical angiographic findings. AFP and hepatitis B surface (HBs) antigen were measured by radioimmunoassay. The size of the primary tumour was evaluated by ultrasonography, computerised tomography and angiography. Size was expressed as the diameter for relatively small and solitary tumours; in the remainder, the proportion of the sum of the tumour areas relative to the whole liver area on the angiogram or computerised tomogram was taken as the size of the tumour as described previously (Nomura *et al.*, 1989). Five hundred patients had liver cirrhosis; 275 patients had histological evidence of cirrhosis, and in the remainder the diagnosis was based on unequivocal clinical grounds (presence of oesophageal varices at endoscopy and or collateral circulation at echography) and laboratory data. The patients were classified into Child's three grades (Child, 1964) based on their clinical status on admission. Furthermore, stratification of the patients was performed according to the staging system described by Okuda *et al.* (1985):

Stage I (not advanced): tumour size less than 50%, no ascites, albumin greater than 3 $g\,dl^{-1}$ and bilirubin less than 3 $mg\,dl^{-1}$.
Stage II (moderately advanced): one or two of the signs of advanced disease present.
Stage III (very advanced): three or all of the advanced signs present.

Surgery, varying from partial resection or enucleation to extended lobectomy, was carried out in 83 patients, transcatheter arterial embolisation using Gelfoam (Upjohn, Kalamazoo, MI, USA) in 128, intra-arterial chemotherapy using a bolus dose or microcapsular forms of mitomycin C and or adriamycin (doxorubicin; Adria laboratories, Colombus, OH, USA) in 166 and other therapeutic methods in 83. No specific treatment was given in 162, who were treated palliatively. Results are given as mean ± standard deviation. Comparisons were made by the chi-square test with Yates' correction. Survival rate was calculated from the time of cancer diagnosis by the life table method, and statistical analysis was performed using the generalised Wilcoxon test. A multivariate analysis of prognostic variables including age, gender, tumour size, bilirubin and albumin values, serum AFP levels and Child's grades was performed. To assess the relative prognostic importance of factors in predicting survival, the Cox proportional hazards regression was employed using a stepwise procedure (Cox, 1972). Calculations were done with the statistical package of SAS as outlined by SUGI Supplemental Library User's Guide (SAS Institute, Carey, NC, USA). $P$-values less than 0.05 were considered significant.

### Results

### *Age and sex distribution of patients*

Figure 1 shows the age and sex distribution of the 622 patients. Of these, 91 or about 15% were 70 years or older. The proportion of females gradually increased as age increased: 4.5% in the $<45$ years group versus 31.8% in the $\geqslant 70$ years group ($P<0.02$). During the same study period, a total of 17,511 patients were admitted to the Department of

Correspondence: F. Nomura, Department of Clinical Pathology, Institute of Medical Science, Tsukuba University 1-1-1, Tennoudai, Tsukuba City, Ibaragi, Japan.
Received 7 January 1994; and in revised form 4 April 1994.

Medicine in our hospital. Among them, the proportions of females in the <45 years group and in the ≥70 years group were quite similar (43.9% *vs* 44.1%), in contrast to the case with patients with HCC.

*Clinical stage of patients and HBsAg prevalence*

Demographic and clinical data on the three age groups of patients are given in Table I. The prevalence of HBsAg-positive cases and the proportion of large tumours (tumour >50% of the liver) was lowest in the elderly group, whereas the distribution of the clinical stages proposed by Okuda was similar among the three age groups. It is of note that HBsAg was positive in nearly 70% of patients younger than 45 years. During the same study period, we cared for a total of 215 patients with post-hepatitic liver cirrhosis in our own unit. The average age of HBsAg-positive patients at the time of diagnosis was significantly lower than that of HBsAg-negative patients (46.8 ± 7.5 *vs* 52.0 ± 4.5, $P<0.001$).

*Therapeutic modalities in HCC patients with various age groups*

Table II summarises the treatments given to patients with HCC in relation to age. Patients aged 70 years or older were more likely to be untreated and were significantly less likely to receive surgery (4/91 *vs* 79/531, $P<0.02$).

**Figure 1** Age and sex distribution (▨, male; □, female) of 622 patients with hepatocellular carcinoma.

**Figure 2** Comparison of survival curves calculated by the life table method between elderly HCC patients (× --- ×) and non-elderly patients (● —— ●) at three different clinical stages. A, stage I; B, stage II; C, stage III.

**Table I** Tumour sizes and clinical stages at the time of diagnosis in 622 HCC patients of various age groups

| *Age (years)* | *n* | *HBsAg (+) (%)* | *Tumour size (%)* ≤5 cm | 5 cm–50% | >50% | *Clinical stage (%)* I | II | III |
|---|---|---|---|---|---|---|---|---|
| <44 | 40 | 67.5 | 9 (22.5) | 11 (27.5) | 20 (50.0)* | 28.2 | 46.1 | 25.7 |
| 45–69 | 491 | 19.1 | 175 (35.6) | 163 (33.2) | 153 (31.2) | 36.0 | 48.6 | 15.4 |
| >70 | 91 | 13.1** | 33 (36.3) | 38 (41.7) | 20 (22.0) | 35.2 | 52.7 | 12.1 |

*$P<0.01$ *vs* >45 years group; **$P<0.01$ *vs* <70 years group.

**Table II** Therapeutic modalities in 622 HCC patients of various ages (percentage in parentheses)

| *Age (years)* | *No. of patients* | *Surgery* | *Intra-arterial chemotherapy* | *Transcatheter arterial embolisation* | *Others* | *No treatment* |
|---|---|---|---|---|---|---|
| <44 | 40 | 8 (20.0) | 9 (22.5) | 4 (10.0) | 9 (22.5) | 10 (25.0) |
| 45–69 | 491 | 71 (14.5) | 132 (26.9) | 104 (21.2) | 63 (12.8) | 121 (24.6) |
| >70 | 91 | 4 (4.4)* | 25 (27.4) | 20 (22.0) | 11 (12.1) | 31 (34.1) |

*$P<0.02$ *vs* <70 years group.

**Table III** Multivariate analysis of major prognostic factors by the Cox proportional hazards model (variables selected in the final model)

| *Variables* | *Regression coefficient* | *P-value* |
|---|---|---|
| Child's grade | 0.64250613 | <0.0001 |
| Tumour size | 0.35645823 | <0.0001 |
| AFP | 0.00000108 | <0.04 |
| Total bilirubin | 0.01762973 | <0.05 |

*Survival of elderly patients with hepatocellular carcinoma*

The median survival of 622 patients regardless of their ages was 13.7 ± 16.8 months as of March 1992. Survival rates for elderly (⩾70 years) and non-elderly (<70 years) patients were compared taking the clinical stage of the tumours into consideration. As shown in Figure 2, there was no statistically significant difference in survival rate between the two age groups for each stage. Furthermore, stepwise Cox regression analysis of the main variables revealed that Child grades, tumour size, total bilirubin and AFP level were significant and independent predictors of survival (Table III), but that age was not.

### Discussion

Various forms of cancer may present different clinical features in the elderly population (Huvos, 1986; Yancik *et al.*, 1986, 1989; Teeter *et al.*, 1987; Walker *et al.*, 1990). In the present study, we assessed the clinical features and survival rates in elderly HCC patients of 70 years of age or older. Analysis of demographic features in these patients revealed that HBsAg positivity was significantly less frequent in elderly than in younger patients. Also, the proportion of females gradually increased with increasing age. The exact reasons for there being a greater proportion of females in the elderly group are not clear, but we speculate that slower progression of chronic viral hepatitis in females and lower prevalence of heavy drinkers may partly account for this.

Stored sera were available for 164 non-A, non-B patients of the present series, and anti-HCV measured by the second-generation enzyme immunoassay (EIA-2, Ortho Diagnostics, Ranitau, NJ, USA) was found in 92%; the age distribution of those 164 patients was similar to that of all the patients included in this study. It has been reported that the elderly are more likely to present at initial diagnosis with advanced stages in the case of ovarian cancer (Yancik *et al.*, 1986), breast cancer (Yancik *et al.*, 1989) and Hodgkin's disease (Walker *et al.*, 1990). However, this was not the case with HCC. Indeed, it was found that the diagnosis was made at a less advanced stage in terms of the size of the tumour in the elderly than in the younger patients (Table I). In Japan, patients with liver cirrhosis are encouraged to have examinations using a combination of real-time ultrasonography and AFP measurement at regular intervals for an early detection of HCC (Okuda, 1986). It is conceivable that the older HCC patients are, the more definite the diagnosis of liver cirrhosis, and as a result they are more likely to undergo such examinations, which, in turn, may lead to early detection of HCC. Increasing age has frequently been reported to have a negative impact on the survival of patients with several types of cancer, such as ovarian cancer (Yancik *et al.*, 1986), breast cancer (Yancik *et al.*, 1989) and Hodgkin's disease (Walker *et al.*, 1990). Again, this was not found to be the case with HCC in this study. The prognosis of the elderly HCC patients was found to be similar to that for the younger cases as assessed by two different analyses. In a stage-matched comparison of elderly and non-elderly patients, the prognosis of the former was not statistically different from that of the latter. Furthermore, in a multivariate analysis using the Cox proportional hazards model age was not selected as an independent negative predictor of survival. The multivariate analyses were also carried out separately in three groups of patients treated by three different modalities (surgery, intra-arterial chemotherapy and TAE), and in each case age was not selected as a significant predictor (data not shown). This observation that old age is not a significant negative predictor of survival in HCC patients is at variance with the findings of other studies (involving relatively small numbers of patients) that suggested advanced age to be associated with a shorter survival in HCC (Chlebowski *et al.*, 1984; Falkson *et al.*, 1988; Calvet *et al.*, 1990; Okada *et al.*, 1992). The exact reason for this discrepancy is not clear, but the difference may be explained in part by the prevalence of advanced cases. Indeed, in those previous studies, the majority of patients, most of whom had systemic chemotherapy, had very advanced disease and their median survivals ranged from only 3.3 to 5.6 months. By contrast, the median survival was 13.7 months in this study. Differences in ethnic background of HCC patients might also account for the differences in survival. The current finding obtained with a large number of patients that old age is not necessarily a negative predictor of survival may be encouraging from a therapeutic point of view. Elderly patients were significantly less likely to undergo surgery (Table II), which is in accordance with a general view that advanced age has an adverse effect on the safe limit of surgical resection. However, percutaneous ethanol injection therapy (Sugiura *et al.*, 1983; Castells *et al.*, 1993), which is definitely less invasive than surgery, is increasingly being carried out and so elderly HCC patients may soon have a better survival with somewhat improved prognostic outcome.

### References

CALVET, X., BRUIX, J., GINES, P., BRU, C., SOLE, M., VILANA, R. & RODES, J. (1990). Prognostic factors of hepatocellular carcinoma in the West: a multivariate analysis in 206 patients. *Hepatology*, **12**, 753–760.

CASTELLS, A., BRUIX, J., BRU, C., FUSTER, J., VILANA, R., NAVASA, M., AYUSO, C., BOIX, L., VISA, J. & RODES, J. (1993). Treatment of small hepatocellular carcinoma in cirrhotic patients: a cohort study comparing surgical resection and percutaneous ethanol injection. *Hepatology*, **18**, 1121–1126.

CHEAH, P.L., LOOI, L.M., LIN, H.P. & YAP, S.F. (1990). Childhood primary hepatocellular carcinoma and hepatitis B virus infection. *Cancer*, **65**, 174–176.

CHILD, III, C.G. (1964). *The Liver and Portal Hypertension*. W.B. Saunders: Philadelphia.

CHLEBOWSKI, R.T., TONG, M., WEISSMAN, J., BLOCK, J.B., RAMMING, K.P., WEINER, J.M., BATEMAN, J.R. & CHLEBOWSKI, J.S. (1984). Hepatocellular carcinoma: diagnostic and prognostic features in North American patients. *Cancer*, **53**, 2701–2706.

COX, D.R. (1972). Regression models and life tables. *J.R. Stat. Soc.*, **34**, 187–220.

DUNK, A.A., SPILIADIS, H., SHERLOCK, S., FOWLER, M.J.F., MONJARDINO, J.P., SCHEUER, P.J. & THOMAS, H.C. (1988). Hepatocellular carcinoma: clinical, aetiological and pathological features in British patients. *Int. J. Cancer*, **41**, 17–23.

FALKSON, G., CNAAN, A., SCHUTT, A.J., RYAN, L.M. & FALKSON, H.C. (1988). Prognostic factors for survival in hepatocellular carcinoma. *Cancer Res.*, **48**, 7314–7318.

HUVOS, A.G. (1986). Osteogenic sarcoma of bones and soft tissues in older persons. A clinicopathological analysis of 117 patients older than 60 years. *Cancer*, **57**, 1442–1449.

LACK, E.E., NEAVE, C. & VAWTER, G.F. (1983). Hepatocellular carcinoma: review of 32 cases in childhood and adolescence. *Cancer*, **52**, 1510–1515.

NOMURA, F., OHNISHI, K. & TANABE, Y. (1989). Clinical features and prognosis of hepatocellular carcinoma with reference to serum alpha-fetoprotein levels. *Cancer*, **64**, 1700–1707.

OKUDA, K. (1976). Clinical aspects of hepatocellular carcinoma–analysis of 134 cases. In *Hepatocellular Carcinoma*, Okuda, K. & Peters, R.L. (eds) pp. 387–436. Wiley: New York.

OKUDA, K. (1986). Early recognition of hepatocellular carcinoma. *Hepatology*, **6**, 729–738.

OKUDA, K., OHTSUKI, T., OBATA, H., TOMIMATSU, M., OKAZAKI, N., HASEGAWA, H., NAKAJIMA, Y. & OHNISHI, K. (1985). Natural history of hepatocellular carcinoma and prognosis in relation to treatment – study of 850 patients. *Cancer*, **56**, 918–928.

OKADA, S., OKAZAKI, N., NOSE, H., YOSHIMORI, M. & AOKI, K. (1992). Prognostic factors in patients with hepatocellular carcinoma receiving systemic chemotherapy. *Hepatology*, **16**, 112–117.

SUGIURA, N., TAKARA, K., OHTO, M., OKUDA, K. & HIROOKA, N. (1983). Percutaneous intratumoral injection of ethanol under ultrasound imaging for treatment of small hepatocellular carcinoma. *Acta. Hepatol. Jpn*, **24**, 920.

TEETER, S.M., HOLMES, F.F. & MCMARLANE, M.J. (1987). Lung carcinoma in the elderly population. Influence of histology on the inverse relationship of stage to age. *Cancer*, **60**, 1331–1336.

WALKER, A., SCHOENFELD, E.R., LOWMAN, J.T., METTLIN, C.J., MACMILLAN, J. & GRUFFERMAN, S. (1990). Survival of the older patients compared with the younger patient with Hodgkin's disease. Influence of histologic type, staging and treatment. *Cancer*, **65**, 1635–1640.

YANCIK, R., RIES, L.G. & YATES, J.W. (1986). Ovarian cancer in the elderly: an analysis of surveillance, epidemiology, and end results program data. *Am. J. Obstet. Gynecol.*, **154**, 639–647.

YANCIK, R., RIES, L.G. & YATES, J.W. (1989). Breast cancer in aging women. A population-based study of contrasts in stage, surgery and survival. *Cancer*, **63**, 976–981.